# The ACEII recombinant *Trichoderma reesei* QM9414 strains with enhanced xylanase production and its applications in production of xylitol from tree barks

**DOI:** 10.1186/s12934-016-0614-4

**Published:** 2016-12-28

**Authors:** Lili Xiong, Ayyappa Kumar Sista Kameshwar, Xi Chen, Zhiyun Guo, Canquan Mao, Sanfeng Chen, Wensheng Qin

**Affiliations:** 1 School of Life Science and Engineering, Southwest Jiaotong University, Chengdu City, 610031 Sichuan Province China; 2 Department of Biology, Lakehead University, Thunder Bay, ON P7B 5E1 Canada; 3 State Key Laboratory for Agrobiotechnology and College of Biological Sciences, China Agricultural University, Beijing, 100193 China

**Keywords:** Xylitol, Tree barks, ACEII gene, Xylanase, *Trichoderma reesei*

## Abstract

**Background:**

ACEII transcription factor plays a significant role in regulating the expression of cellulase and hemicellulase encoding genes. Apart from ACEII, transcription factors such as XYR1, CRE1, HAP2/3/5 complex and ACEI function in a coordinated pattern for regulating the gene expression of cellulases and hemicellulases. Studies have demonstrated that ACEII gene deletion results in decreased total cellulase and xylanase activities with reduced transcript levels of lignocellulolytic enzymes.

**Results:**

In this study, we have successfully transformed the ACEII transcription factor encoding gene in *Trichoderma reesei* to significantly improve its degrading abilities. Transformation experiments on parental strain *T. reesei* QM9414 has resulted in five genetically engineered strains *T*/Ace2-2, *T/*Ace2-5, *T*/Ace2-8, *T*/Ace5-4 and *T/*Ace10-1. Among which, *T*/Ace2-2 has exhibited significant increase in enzyme activity by twofolds, when compared to parental strain. The *T*/Ace2-2 was cultured on growth substrates containing 2% bark supplemented with (a) sugar free + MA medium (b) glucose + MA medium and (c) xylose + MA medium. The bark degradation efficiency of genetically modified *T*/Ace2-2 strain was assessed by analyzing the xylitol production yield using HPAEC. By 6th day, about 10.52 g/l of xylitol was produced through enzymatic conversion of bark (2% bark + MA + xylose) by the *T*/Ace2-2 strain and by 7th day the conversion rate was found to be 0.21 g/g. Obtained results confirmed that bark growth medium supplemented with d-xylose has profoundly increased the conversion rate of bark by *T*/Ace2-2 strain when compared to sugar free and glucose supplemented growth media. Results obtained from scanning electron microscopy has endorsed our current results. Bark samples inoculated with *T*/Ace2-2 strain has showed large number of degraded cells with clearly visible cavities and fractures, by exposing the microfibrillar interwoven complex.

**Conclusion:**

We propose a cost effective and ecofriendly method for the degradation of lignocellulosic biomass such as bark to produce xylitol by using genetically modified *T. reesei*. Efficient conversion rate and production yield obtained in our current study provides a great scope for the xylitol industries, as our method bypasses the pretreatment of bark achieving clean and low-cost xylitol production.

## Background

Xylitol a naturally occurring five-carbon sugar alcohol, popularly known as a low-calorie sugar substituent. The sweetness of xylitol is thrice that of mannitol, twice that of sorbitol and equal to sucrose with only two-thirds calories of sucrose. Insulin independent metabolism of xylitol makes it a significant alternative for the sucrose thus, it is highly used as sugar substituent for diabetic patients [1]. Commercially, xylitol is being used as sweetener in chewing gums, as bacteria cannot ferment xylitol. It was also reported that xylitol maintains the acid–base balance and prevents the formation of dental caries and oral cavities [2]. Zabner et al. [3], have reported that xylitol prevents bacteria from binding to the human cells thus, protecting us from respiratory tract infections [3]. In addition, xylitol can promote the intestinal absorption of calcium, reduce bone loss, maintain normal bone density, reduce the activity of the liver transaminase etc. Due to its wide range of applications in medicine and food sectors, there is a tremendous demand for xylitol production, it was expected that by 2020, global consumption of xylitol will reach to 242 thousand metric tons with a market value of 1 billion US$ [4].

**Fig. 1:**
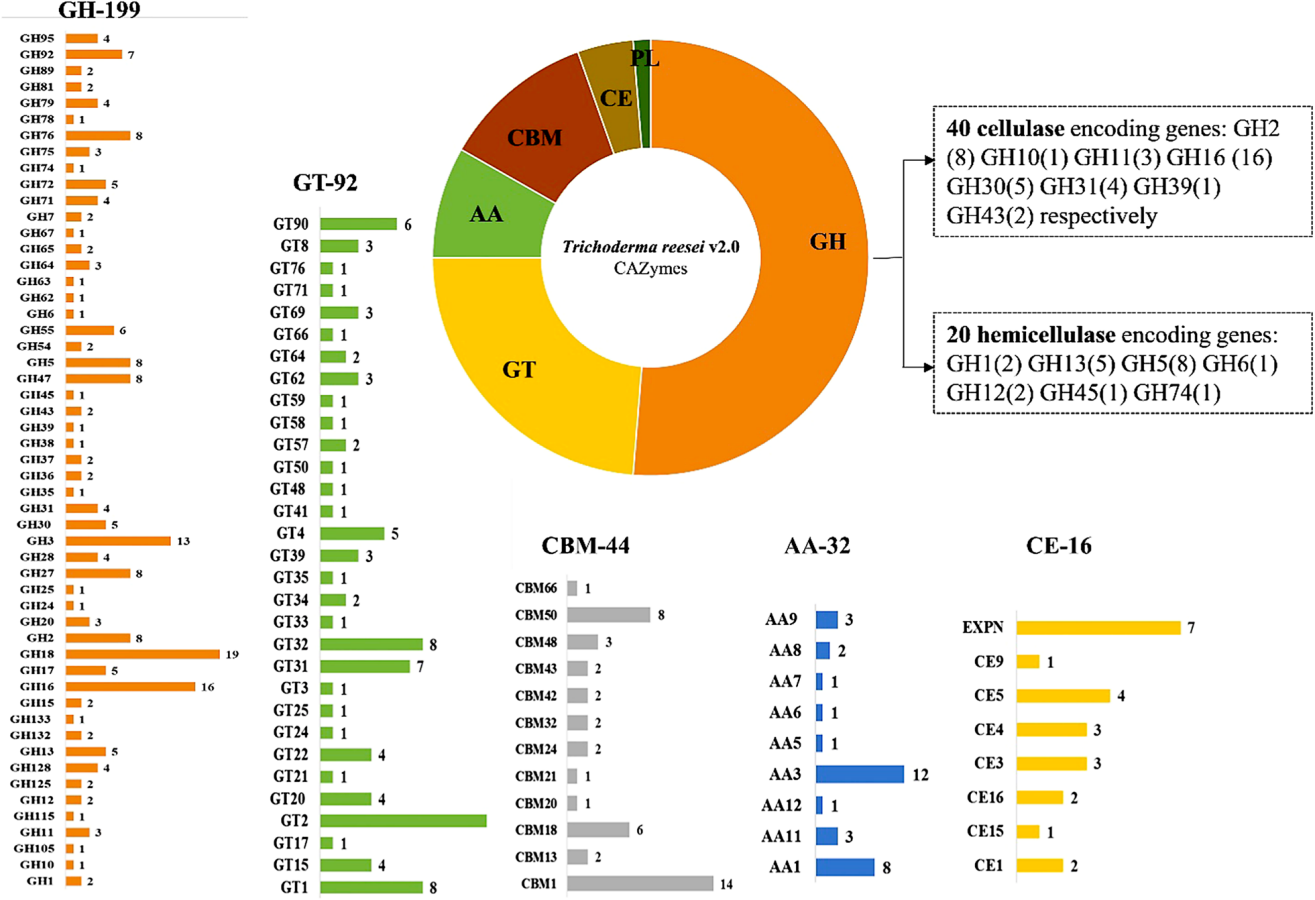
Illustrated representation of *T. reesei* v2.0 CAZymes distribution with a special note on cellulase and hemicellulase encoding genes, GH (glycoside hydrolases), GT (glycosyl transferase) CBM (carbohydrate binding modules) auxiliary activities (AA) and CE (carbohydrate esterases), and corresponding number on top of *bar graph* represents total number of genes encoding for each class of CAZymes [13]

Naturally, xylitol occurs in various fruits and vegetables in lower proportions, making its extraction difficult and expensive. Intricate units of lignocellulose present in the plant biomass attracts various research groups around the world for the production of xylitol, as it comprises 20–35% of hemicellulose [4]. Xylan rich xylose or hemicellulose hydrolysate can be reduced to xylitol by either chemical or microbiological methods. Catalytic hydrogenation method is the major chemical method used in industries for the production of xylitol, however this method requires hydrogen for the conversion of d-xylose to xylitol. The catalytic hydrogenation has several disadvantages such as (a) it is performed under high temperature and pressure conditions, (b) complicated process, (c) poor security (d) causes environmental pollution, (e) this process requires pure xylose as raw material, (f) conversion ratio of xylose to xylitol is only 50–60%. These disadvantages has raised the scope around the world for the microbial production of xylitol [5]. Xylan the major constituent of hemicellulose is initially pretreated by chemical and enzymatic hydrolysis methods, producing xylose which is further enzymatically converted to xylitol using xylose reductase a NADPH dependent enzyme secreted by microorganisms [6, 7]. Thus, efficient degradation of xylan is one of the key steps in the process of xylitol production. Microorganisms secrete various xylan degrading glycoside hydrolases such as endo-1, 4-β-d-xylanase (endoxylanase), β-xylosidase etc., and carbohydrate esterase such as acetyl xylan esterase and ferulic acid esterase. Among various glycoside hydrolases secreted by microorganisms, endoxylanase play a significant role in hydrolysis of xylan [8, 9] thus, improving total xylanase enzyme activity is the hot topic in the field of xylitol production in the recent years. Various microorganisms such as *Aspergillus* spp*., Trichoderma* spp., *Penicillium* spp., *Bacillus* spp. etc. have been found to secrete xylanases. *Trichoderma reesei* is one of the highly studied and commercially used fungi for its cellulase and hemicellulase activities [10–12]. Martinez et al. [13], have sequenced and assembled the whole genome sequence of *T. reesei* for the first time [13]. The 34 Mb whole genome sequence of *T. reesei* was assembled into 89 scaffolds with 9129 predicted gene models. *T. reesei* v 2.0 genome codes for wide range of lignocellulolytic CAZymes which are classified into 199 glycoside hydrolases (GH), 92 glycosyl transferases (GT), 44 carbohydrate binding modules (CBM), 16 carbohydrate esterases (CE) and five polysaccharide lyase (PL) (Fig. 1) [13].

The enzymatic saccharification of cellulose by *T. reesei* involves a synergistic mixture of different cellulolytic enzymes. The *T. reesei* cellobiohydrolase (CBH1) was the first eukaryotic cellulolytic enzyme to be cloned and structurally analyzed [14]. Several genomic and transcriptomic studies have been conducted on *T. reesei* to reveal the functional proteomics of the cellulolytic and hemicellulolytic enzymes. Various gene products encoding for the *T. reesei* lignocellulolytic enzymes are coordinately regulated by set of transcriptional factors, such as XYR1, ACE1, ACE2, CRE1 and HAP 2/3/5 [15], which are required for the positive and negative regulation of cellulolytic and hemicellulolytic enzymes [16, 17]. Among these transcription factors, carbon catabolite repressor (CRE-1) is the master regulator, it was reported that CRE1 completely stops the transcription of the target genes when simple carbon substrates like glucose are present in the medium [14]. Molecular studies were conducted on *T. reesei* QM6a for the mutagenesis of CRE-1 gene for the overexpression of the cellulolytic enzymes, which has resulted in *T. reesei* strain RUT-C30 with higher basal levels and increased rate of cellulase production [18–20]. According to Zou et al. [21], CRE1 binding motifs can be replaced in the promoter regions of cellobiohydrolase with the well-known cellulase activators, which reduces the CRE1 and increases the expression of cellobiohydrolase under different conditions [21]. Similar to the CRE1, ACE1 transcription factor is also a C2H2 zinc finger repressor factor, and ACE1 gene deletion significantly improves the expression of both cellulolytic and hemicellulolytic enzymes [22]. Xylanase regulator 1 (XYR1) is a zinc binuclear cluster (Zn(II)_2_Cys6) protein, strictly regulates the expression of various lignocellulolytic enzymes by binding to the GGCTAA-motif, gene deletions experiments of *xyr*1 shuts down the cellulase and hemicellulase encoding genes [17, 23]. Finally, transcription factors such as ACE1, ACE2 and ACE3 are responsible for the regulation of cellulase and hemicellulase encoding genes in *T. reesei* [22, 24, 25]. Similar to XYR1, ACE2 and ACE3 transcription factors are zinc binuclear cluster proteins. ACEII gene (*ace2*) is a positive transcription regulator of genes encoding for xylanases and various cellulolytic enzymes. For the first time Aro et al. [24], has performed the ACE2 gene knock out experiments on *T. reesei*, which has resulted in decreased mRNA levels of cellulase and xylanase encoding genes such as *cbh1, cbh2, egl1, egl2* and *xyn* respectively, and reducing the total cellulase activity by 30–70% [24] (Table 1).

**Table 1:** Lists the set of transcription factors required for the positive and negative regulation of lignocellulolytic enzymes in *Trichoderma reesei*

Transcription factor	Structure	Consensus sequence	References
Positive regulators in *T. reesei*			
XYR1	Zinc binuclear (Zn(II)_2_Cys6) cluster protein	5′GGCTAA-3′	[26, 27]
ACE2	Zinc binuclear (Zn(II)_2_Cys6) cluster protein	5′GGCTAATAA-3′	[26, 27]
ACE3	Zinc binuclear (Zn(II)_2_Cys6) cluster protein	5′GGCTAA-3′	[14]
HAP2/3/5	Multimeric protein complex	5′CCAAT-3′	[26, 27]
Negative regulators in *T. reesei*			
ACE1	C2H2 (Cys2-His2) zinc finger type transcription factors	5′ AGGCA-3′	[27]
CRE1	C2H2 (Cys2-His2) zinc finger type transcription factors	5′SYGGRG-3′	[27, 28]

According to Häkkinen et al. [25], overexpression of ACE3 gene showed a higher transcription levels of cellulolytic and hemicellulolytic encoding genes [25]. The overexpression experiments on ACE-2 gene has not been conducted yet [14]. The zinc binuclear cluster proteins of *T. reesei* XYR1, ACE2 and ACE3 are similar to Gal4 protein of *Saccharomyces cerevisiae,* the Gal 4 protein along with Gcn5 containing SAGA complex, promotes the transcription of target genes by histone acetylation and euchromatin formation [14, 29, 30]. According to Xin et al. [31], the Gcn5 orthologous gene in *T. reesei* play a crucial role in the cellulase expression as it is required for the histone acetylation of *cbh1* promoter [31].

Naturally, the polysaccharides present in the plant cell wall are acetylated making them weak acids [32–34]. Along with these acetylated saccharides plant cell wall also contains furan and phenolic derivatives which makes the process of hydrolysis and fermentation difficult by the plant biomass degrading microorganisms [34, 35]. Thus, to achieve high production yields of bioproducts it is highly necessary to separate intricate plant cell wall structure for obtaining mono or oligosaccharides. Which can be achieved by biorefinery and biofuel industries by developing a consolidated process involving pretreatment, hydrolysis, fermentation and purification steps [34]. Conventionally several pretreatment methods were developed such as hydrothermal pretreatment (autohydrolysis, steam explosion, steam extrusion), wet oxidation, acid hydrolysis, alkaline hydrolysis, Organosolv pretreatment and pulsed electric field pretreatment etc. Above mentioned pretreatment methods where already extensively studied and reviewed, few where listed here [34, 36–39]. Alves et al. [40], have used different bases and acids by changing the pH level of the hydrolysate, to decrease the total concentration of toxic compounds present in the sugarcane bagasse hydrolysate [40]. Therefore, pretreatment step plays a crucial role in biofuel and biorefining industries for the biological degradation and conversion of plant biomass. However, pretreatment methods are not cost effective and pose risk to the environment and studies have showed that several parameters need to be considered for the usage of pretreatment methods [41]. Thus, developing genetically efficient microbial strains for the degradation of plant biomass through bypassing conventional pretreatment methods are highly necessary in the present era.

In this study, we have constructed an expression vector *pace2*-*hph*-*PRIM* for the overexpression of ACE2 gene in *T. reesei.* Several studies have already reported on utilization of tree barks for the production of value added products, as it contains good amounts of cellulose and hemicellulose, thus we have proposed a cost effective and eco-friendly method (as it bypasses the pretreatment steps) for the efficient production of xylitol from tree bark [42].

## Methods

### Reagents, plasmids and primers

The solutions used in this study were prepared with ultra-pure grade water purified with Millipore device. The chemicals used in our study were obtained from manufacturers Sigma-Aldrich, Fischer Scientific, Bioshop, VWR, FroggaBio and BioRad. All the primers used in the present study were synthesized by Eurofins MWG Operon company. The detailed information about the plasmids and primers used in the present study were listed in Tables 2 and 3 respectively.

**Table 2:** Strains and plasmids

Plasmids	Genotype and description	Source
*pTWO*	5775 bp, containing *hygromycin* B resistance expression cassette (hph), used to construct expression vectors	Provided by Dr. Bernhard Seiboth, Institute of Chemical Engineering, Vienna University of Technology Vienna, Austria
*pPRIMex30*	5439 bp, containing hygromycin B resistance expression cassette (hph), used to construct expression vectors	Provided by Dr. Bernhard Seiboth, Institute of Chemical Engineering, Vienna University of Technology Vienna, Austria
*Pace*-*hph*-*PRIM*	7139 bp, recombinant containing ace2 gene controlled by pgk1 promoter and ace 2 terminator	This work

**Table 3:** Primers

Name	Sequence (5′→3′)	Restriction enzyme	Length (bp)
Ace2-P8	5′-TATTCTAGAATGGACCTCCGGCAAGCATGT-3′	*Xba*I	30
Ace2-P6	5′-GCAAAGCTTTCGTCTGTTTTTGATGACTTC-3′	*Hin*dIII	30
hph-P1	5′-GCGAAGCTTGAGAGCTACCTTACATCAAT-3′	*Hin*dIII	29
hph-P2	5′-GTGAAGCTTATACCCCAGTCCAGATCATG-3′	*Hin*dIII	29
pki1-P1	5′-GACGAAGACCTGACTCGTGA-3′	*Xba*I, *Eco*RI	20

**Table 4:** Different growth mediums used in the present study

Growth medium	Composition (g/l)	pH
Potato dextrose agar	Potato dextrose 15 g, sugar 20 g, KH_2_PO_4_ 3 g, MgSO_4_·7H_2_O 2 g, agar 15 g	pH is maintained between the range of 5.6–5.8
Yeast glucose medium	Yeast extract 5 g, glucose 20 g, microelement solution of 400 μl. The microelement solution consists of EDTA 10 g, ZnSO_4_·7H_2_O 4.4 g, MnCl_2_ 1.01 g, CoCl_2_·6H_2_O 0.32 g, CuSO_4_·5H_2_O 0.315 g, (NH_4_)_6_Mo_7_O_24_·5H_2_O 0.22 g, CaCl_2_·2H_2_O 1.47 g, FeSO_4_·7H_2_O 1 g, dissolved in 900 ml double distilled water	The pH is adjusted to 6.0 using 1 M NaOH, once all the constituents are dissolved add 1 M HCl to adjust the pH to 4.0, later the solution is made up to 1000 ml using dd-H_2_O
Mandel and andreotti (MA-medium)	KH_2_PO_4_ 2 g, (NH_4_)_2_SO_4_ 1.4 g, Urea 0.3 g, FeSO_4_·7H_2_O 0.005 g, MnSO_4_·H_2_O 0.0016 g, ZnSO_4_·7H_2_O 0.0014 g, CoCl_2_ 0.002 g, MgSO_4_·7H_2_O 0.3 g, CaCl_2_ 0.3 g, peptone 0.75 g, glucose 10 g	pH is maintained between the range of 4.8–5.0
2% glycerol-MA medium	Glycerol 10 g in 500 ml of MA medium	pH is maintained between the range of 5.4–5.8
2% bark-MA sugar-free medium	Bark powder 10 g dissolved in 500 ml of MA medium (composition is same as mentioned above but *without glucose*)	pH is maintained between the range of 5.4–5.8
2% bark-MA glucose medium	Bark powder 10 g dissolved in 500 ml of MA medium with glucose 10 g	pH is maintained between the range of 5.4–5.8
2% bark-MA xylose medium	Bark powder 10 g dissolved in 500 ml of MA medium with xylose 10 g (glucose is replaced with xylose)	pH is maintained between the range of 5.4–5.8
Minimal salt medium (MSM) consists	Glucose 20 g, (NH_4_)_2_SO_4_ 5 g, KH_2_PO_4_ 15 g, MgSO_4_·7H_2_O 0.6 g, CaCl_2_ 0.6 g, FeSO_4_·7H_2_O 0.005 g, MnSO_4_·H_2_O 0.0016 g, ZnSO_4_·7H_2_O 0.0014 g, CoCl_2_ 0.002 g	pH between the range of 5.0–5.5
LB-medium	Tryptone 10 g, yeast extract 5 g, NaCl 5 g	pH is adjusted to 7.0

### Microbial strains and culture mediums

The *T. reesei* QM9414 (ATCC26921) mutant strain used as the host for the *ace*2 gene transformation experiment, *T. reesei* QM9414 was gifted by Dr. Tianghong Wang, Shandong University, China. *Escherichia coli* JM109 strain was used for the construction and propagation of vector. *T. reesei* QM9414 strains were cultured and maintained on potato dextrose agar medium. PDA growth medium supplemented with 50 µg/ml hygromycin used for screening the *T. reesei* transformants. Yeast glucose medium (YG) supplemented with 50 µg/ml of hygromycin B [43] was also used for isolation of positive *T. reesei* transformants. The recombinant *T. reesei* strains were cultured on different carbon sources with final concentration of 2% (w/v) such as 2% glycerol, 2% bark-sugar free medium, 2% bark-glucose medium, 2% bark-xylose medium supplemented with Mandel and Andreotti (MA-medium) [44]. The composition of the above-mentioned growth mediums was shown in Table 4. The bark used in the growth medium were obtained from Resolute Forest Products, Thunder Bay, Canada, the composition of the bark was: 65% Jack Pine (*Pinus banksiana*), 25% Black Spruce *(Picea mariana*), 3–5% Balsam Fir (*Abies balsamea*) and some Aspen (*Populus tremuloides*) hardwood.

### Recombinant plasmid pace2-hph-PRIM construction

The 2.7 kb of *ace2* transcription factor sequence of *T. reesei* was retrieved from the NCBI Gene database. The PCR primers Ace2-P8 and Ace2-P6 with restriction enzyme cutting sites *Xba*I and *Hin*dIII (Table 3) were designed based on the *ace*2 coding sequence of *T. reesei*. The genomic DNA of *T. reesei* QM9414 was isolated using the Norgen Fungi/yeast DNA extraction kit, the genomic DNA isolated was used as template for PCR amplification. The *ace*2 gene fragments obtained from PCR amplification were ligated with T-cloning vector, these ligated plasmids were further transformed into *E. coli* JM109 competent cells, and these cells were cultured on LB growth medium containing ampicillin for screening the positive transformants. The plasmid DNAs from positive transformants were extracted using the Norgen plasmid miniprep kit, and was digested using the restriction enzymes *Xba*I and *Hin*dIII. Gene fragments obtained as a result of restriction were purified and ligated with linearized DNA of pPRIMex30 vector (digested with *Xba*I and *Hin*dIII), these ligated vectors were again transformed into *E. coli* JM109 competent cells through electroporation. The *E. coli* JM109 competent cells containing recombinant plasmid pPRIMex30-ace2 were cultured on LB growth medium with ampicillin used for screening positive transformants, DNA from recombinant plasmid pPRIMex30-ace2 was isolated same as mentioned above. The recombinant plasmid pPRIMex30-ace2 was digested with *Hin*dIII and dephosphorylated using SAP after purification. The plasmid pTWO containing *hph* gene expression cassette was used as template for amplifying hph gene using the primers hph-P1 and hph-P2 (with *Hin*dIII recognition sites). PCR fragments obtained were further digested with *Hin*dIII restriction site and ligated with the linearized pPRIMex30-ace2 vector DNA. Followed by transferring 1 μl of ligated product into the *E. coli* JM109 competent cells, the pace2-hph-PRIM plasmid DNA from the positive transformed cultures were collected and subjected to digestion verification using the *Hin*dIII restriction sites, followed by analyzing the fragments on 1% agarose gel.

### Screening ace2 recombinant *T. reesei* strains using colony PCR

The recombinant plasmid pace2-hph-PRIM containing *ace2* transcription factor and hygromycin B phosphotransferase (*hph*) expression cassette (used as a selection marker), was transformed into *T. reesei* QM9414 strain. The protoplasts from the *T. reesei* QM9414 strain was isolated and transformed with pace2-hph-PRIM plasmid DNA based on the methods proposed by Szewczyk et al. [45]. The positive transformants were isolated by culturing the *T. reesei* QM9414 strains on PDA plates supplemented with 50 μg/ml of hygromycin at 30 °C for 3–4 days. On 4th day 40 positive clones from the PDA-hygromycin plates were transferred to yeast glucose growth medium supplemented with hygromycin B and cultured at same growth conditions. From the resulting positive cultures, sixteen clones were randomly picked for the extraction of the plasmid DNA. The plasmid DNA isolated was further confirmed by PCR amplification using *pki1*-P1 and *ace*2-P6 primers (Table 3), thus obtained PCR gene fragments were analyzed using 1% agarose gel electrophoresis.

### Characterization of recombinant *T. reesei* ace2 strain

The spores from the *T. reesei* positive transformants containing *ace*2 gene was collected from the yeast glucose-hygromycin B growth medium. The spores from the cultures were diluted at 10^3^, 10^4^, 10^5^/ml concentrations, 100 µl of each dilution was taken and spread on to the PDA agar plates and cultured at 30 °C for 18 h. The spores were directly observed using the microscope in the aseptic condition until the spores started to produce mycelia. Then a single spore was cut from agar medium using a sterile scalpel and was transferred into PDB agar plate containing 50 μg/ml of *Hygromycin* B. The plates were incubated at 30 °C until the whole plate was covered by new mycelium. The single colony picked from the plate was used for DNA extraction. The positive colony was identified by PCR using the primers of pki1-p1 and Ace2-P6 (Table 3), later the gene fragments were further analyzed using 1% agarose gel electrophoresis.

### Filter paper assay (FPA)

The total cellulase activity was performed using filter paper assay (FPA), as described previously [46]. Initially, 200 μl of *T. reesei* spore suspensions with a final concentration of 1 × 10^8^/l, was inoculated into 50 ml of MS medium supplemented with 2% Avicel and incubated at 30 °C, 180 rpm for 7 days. The culture samples were collected every 24 h and these samples were further used for FPA activity by centrifuging the samples for 2 min at 13,000 rpm. The assay mixture consists of 6 mm diameter Whatman No. 1 filter paper with average weight of 3.0 mg (ThermoFischer Scientific, Canada) in 50 mM sodium acetate buffer (pH 4.8) and 20 μl of culture filtrate. The reaction mixture is transferred to 96-well plate with lid and incubated at 50 °C water bath, after 6 min the microplate is cooled down to room temperature. The reducing sugars obtained from the above assay was measured using the DNS reagent (3,5-dinitro salicylic acid), reducing sugars released during the assay was considered as glucose equivalents. The wells containing only sodium citrate buffer and filter paper can be used as substrate control, culture filtrate and buffer without filter paper acts as enzyme control, assay was performed in triplicates. The microplate containing both the test and control samples were placed in a boiling water bath with a lid, after 5 min the microplate is cooled to room temperature and the concentration of the reducing sugar was measured using a spectrophotometer at an absorbance of 540 nm. The FPU/ml was calculated based on the equation previously reported by Xiao et al. [47], based on which FPU/mg was calculated using the protein concentration respectively. One filter paper unit is defined as average of µmol of glucose (reducing sugar) equivalents released per min in the reaction mixture. The glucose standard curve was performed at different concentrations ranging from 0, 2, 4, 6, 8 and 10 mg/ml respectively. The reaction mixture consists 20 μl glucose solution of above mentioned concentrations and 120 μl of DNS solution. The reaction mixture was transferred to a 96-well plate with lid and placed in a boiling water bath. After 5 min the microplate was removed and cooled down to attain the room temperature. The concentration of glucose was measured using a spectrophotometer at an absorbance of 540 nm, with 0 mg/ml as control. The glucose standard curve was plotted with glucose concentration (mg) on x-axis and O. D values on Y-axis.

$$\begin{aligned} {\text{Enzyme activity}}\;\left( {{\text{FPU}}/{\text{ml}}} \right) = \frac{{({\text{Sample }}\, A_{540} )}}{{({\text{Glucose solution }}A_{540} /{\text{mg}})}}\left( {5.55\,{\upmu \text{mol/mg}}} \right) \\ \quad \times \left( {\frac{1}{{60\,{ \text{min} }}}} \right)\left( {\frac{1}{{0.02\,{\text{ml}}}}} \right) \end{aligned}$$

### Determination of xylanase enzyme activity

The xylanase enzyme activity was performed using DNS reagent. Initially, 200 μl of *T. reesei* spores at a concentration of 1 × 10^8^/l was taken and inoculated in 50 ml MA medium supplemented with 2% glycerol liquid medium and incubated at 30 °C at 180 rpm for 7 days. About 500 μl culture samples were recovered every 24 h, these retrieved sample solutions were centrifuged at 13,000 rpm for 2 min and thus obtained culture supernatant is used for the xylanase enzyme activity using DNS reagent. The reaction mixture contained 1% birch wood xylan in 50 mM sodium citrate buffer solution (pH 4.8), 25 μl of culture supernatant and 150 μl of DNS reagent. The enzyme reaction is stopped by adding DNS reagent, the assay was performed in 96-well plate with a lid. The microplate is placed in a boiling water bath, after 5 min the microplate is cooled down to room temperature and the xylanase activity was measured using a spectrophotometer at an absorbance of 570 nm. The substrate control consists of 25 μl of buffer solution with 1% birch wood and DNS reagent, enzyme control consists of 25 μl culture supernatant and buffer solution along with DNS reagent. The assay for the test and control samples were performed in triplicates. The xylanase standard curve was performed using different concentrations of xylan solutions ranging from 0, 2, 4, 6, 8 and 10 mg/ml respectively. The reaction mixture contains 25 μl xylan solution, 50 mM of sodium citrate buffer solution (pH 4.8), 25 μl of culture supernatant and 150 μl of DNS solution, the assay was performed in 96-well microplate. The microplates containing reaction mixture is placed in water bath at 50 °C, after 5 min the microplates were cooled down to room temperature. The xylanase activity was measured by a spectrophotometer at an absorbance 570 nm. The xylanase standard curve was plotted with xylan concentration (mg) on x-axis and the O. D values on the y-axis.

### Xylitol determination using HPAEC

The conversion yields of xylitol from bark by *T. reesei* QM9414 and transformed strains were estimated using HPAEC. Initially, *T. reesei* strains were inoculated on PDA medium and incubated for 7 days at 30 °C. After 7th day, the green color spores from the cultures were taken and filtered using 5 ml sterile solution, the above solution is filtered using 12 layers of lens paper. The filtered spores of 1.0 × 10^7^/l concentration were inoculated in 2% glycerol MA medium and incubated at 30 °C, 180 rpm for 48 h. On third day, mycelium was isolated from the 2% glycerol MA medium and washed with MA medium to remove the residual glycerol, followed by transferring about 1 g of mycelium into 50 ml of 2% bark-sugar free MA medium, 2% bark-glucose MA medium and 2% bark-xylose medium respectively. About 500 μl of samples were taken from the above cultures every 24 h, these samples were initially centrifuged at 16,000 rpm for 5 min and the culture supernatants were stored at 4 °C. The above collected samples were used for xylitol measurement, initially from the collected samples about 500 μl were filtered using MinisartRC 4 filter to remove fungal spores and other particles. The culture supernatant was diluted using the methanol containing 2 mM NaOH solution, these samples were analyzed using High-Performance Anion Exchange Chromatography with Pulsed Amperometric Detection (HPAE-PAD) using a Dionex ICS3000 system equipped with a 3 × 150 mm CarboPac PA20 Carbohydrate Column and Guard. 52 mM NaOH (isocratic) eluent was used at a flow rate of 0.5 ml/min with a full loop injection volume of 25 μl. The column was maintained at 30 °C, and a gold (Au) electrode with quadruple potential was used for separation.

### Scanning electron microscopy analysis of T/Ace2-2

The scanning electron microscopy (SEM) analysis was performed to understand the effect of transformed *T. reesei ace*2-2 strains on the bark samples. The spores from T/Ace2-2 strains with a concentration of 1.0 × 10^7^/l were initially inoculated on bark samples. The Ace2-2 inoculated bark samples were placed in 50 ml MA medium, and incubated at 30 °C for 14–28 days at 200 rpm. The un-inoculated bark samples placed in MA-growth medium can be used as control samples. After 28 days, the samples were immersed in 2% glutaraldehyde (in 0.1 M phosphate buffer solution with pH 7.2) at 4 °C. After 2 h, bark samples were washed with 0.1 M phosphate buffer for three times (10 min each wash), further these samples were dehydrated using a range of ethanol solutions from 50, 70, 80, 90 and 100%, each dehydration step was performed for 10 min. Finally, the bark samples were cooled to room temperature and coated with gold for 45 s in a Denton-DeskII sputter coater (Denton Vacuum USA, Moorestown, NJ). Thus, obtained samples were examined under scanning electron microscope (Hitachi SU-70, Japan) at 5 kv.

## Results

### Construction of expression vector ace2-hph-PRIM

**Fig. 2:**
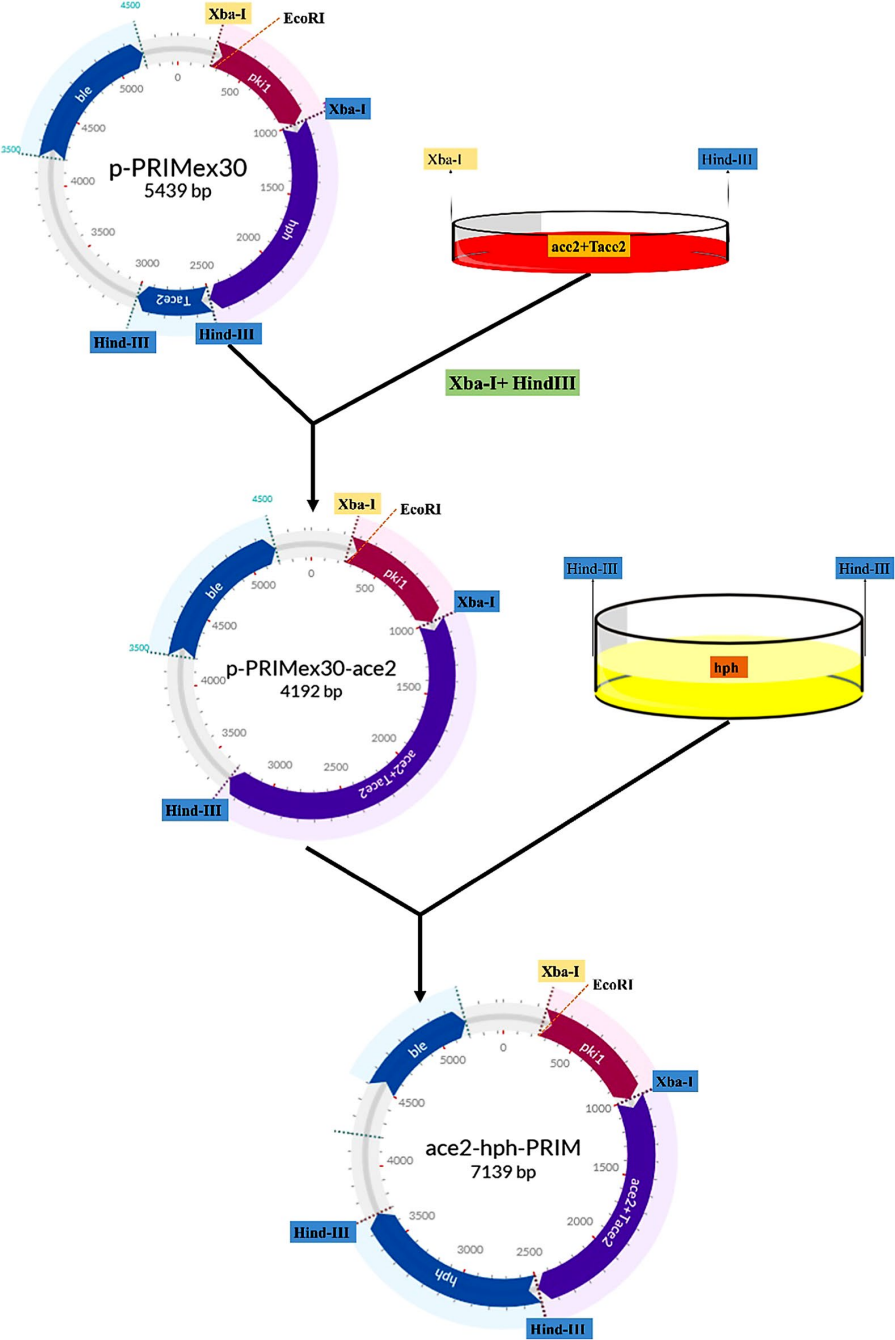
Pictorial representation of construction and expression of pace2-hph-PRIM vector (plasmid map) in *T. reesei*

In order to increase the production of xylanase and overall cellulolytic and hemicellulolytic activities of *T. reesei,* we have constructed and overexpressed *ace*2 gene incorporated in an expression vector pace2-hph-PRIM. Earlier studies have reported that cellulase encoding genes are regulated by a set of transcription factors a) positive regulators XYR1, ACE2 and HAP2/3/5 b) repressors ACE1 and CRE1. XYR1 (xylanase regulator 1) is the main activator for the cellulase and hemicellulase encoding genes as the XYR1 gene deletion resulted in impaired induction of cellulases and hemicellulases [16, 17]. Similarly, deletion if ACE2 gene resulted in decreased expression of cellulases and reduced the overall cellulase activity by 30–70%. The expression of the xylanase encoding genes were significantly affected by the *ace*2 gene deletion resulting in lowered expression of xyn2 gene [24]. It was also reported that XYR1 and ACE2 binds to the same promoter motif sequences [GGC (T/A)_4_] [24, 48]. The ACE2 gene was selected for the over expression of cellulolytic and hemicellulolytic coding genes in *T. reesei* QM9414. Portnoy et al. [49], have performed the regulation of expression of activator genes such as *xyr1*, *ace2* and corepressor *ace1* genes, involved in the cellulase biosynthesis mechanisms in the presence of lactose inducer by *T. reesei* QM9414 [49]. This study has reported that *xyr1*, *ace2* and *ace1* genes are induced by d-galactose, though the gene induction which is independent of d-galactose metabolism, elevation of basal transcription levels of *xyr1* and decreased gene expression of *ace1* by lactose is significantly involved in generation of hyper producing strains, thus *xyr1, ace2* and *ace1* genes highly control cellulase production [49]. Mach-Aigner et al. [50], has conducted a study to demonstrate the induction of xylanase gene expression upon concentrations of d-xylose, this study has reported that higher concentrations of d-xylose resulted in decreased xylanase gene expression and also proved the antagonistic role of carbon catabolite repressor 1(CRE1) in d-xylose dependent gene induction [50]. Thus, strong involvement of ACE2 gene in the expression of cellulolytic and hemicellulolytic genes was the reason for the present overexpression study of ACE2 gene. The plasmid construction and expression map for pace2-hph-PRIM was reported in Fig. 2.

**Fig. 3:**
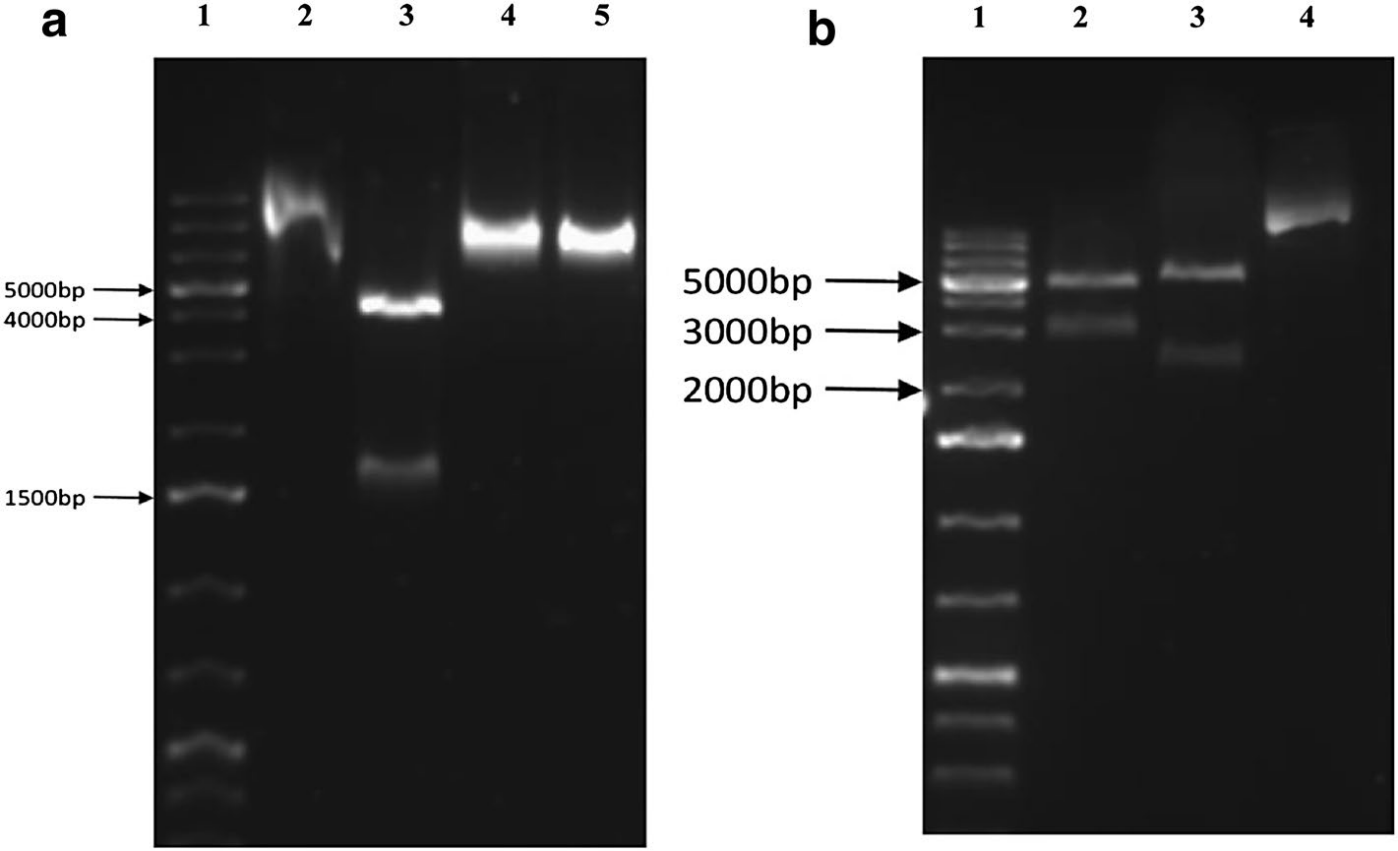
**a** Restriction analysis of plasmid pPRIMex30-ace2 showing, *Lane 1* 1 kb Plus DNA Ladder; *Lane 2* pPRIMex30-ace2; *Lane 3* pPRIMex30-ace2/*Xba*I + *Hin*dIII; *Lane 4* pPRIMex30-ace2/*Xba*I; *Lane 5* pPRIMex30-ace2/*Hin*dIII. **b** Restriction analysis of plasmid pace2-hph-PRIM showing, *Lane 1* 1 kb Plus DNA Ladder; *Lane 2* pace2-hph-PRIM/*Xba*I; *Lane 3* pace2-hph-PRIM/*Xba*I; +*Hin*dIII; *Lane 4* pace2-hph-PRIM

**Fig. 4:**
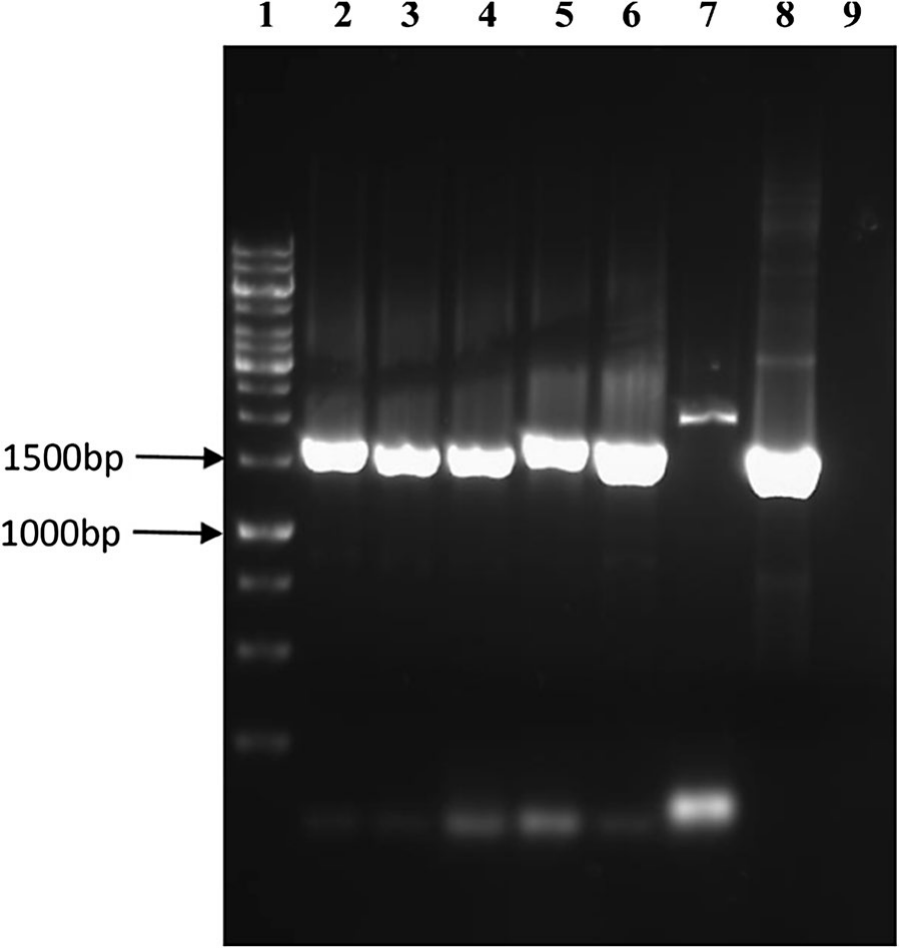
Shows the electrophoresis image of PCR amplified products of *ace2* showing; *Lane 1* 1 kb DNA marker; *Lane 2*–*6* ace2 PCR amplified products of *T. reesei ace2* recombinant strains; *Lane 7* PCR amplification with genomic DNA of *T. reesei* QM9414 as a negative control. *Lane 8* PCR amplification using plasmid of pace2-hph-PRIM as a positive control

The PCR quality *T. reesei* QM9414 genomic DNA was used as template for the isolation of *ace2* gene using *ace2*-P6 and *ace2*-P8 primers (Table 3), amplified PCR products resulted in 1.4 kb band on 1% agarose gel electrophoresis, separated band was further retrieved and sequenced. The 1411 bp long sequence obtained from sequence analysis was analyzed using NCBI BLAST, which showed 100% homology with the *ace2* gene sequence of the *T. reesei* (from initiation site to downstream terminator region). The *ace2* gene fragment obtained from PCR was further transferred to pPRIMex30 through double digestion using *Xba*I and *Hin*dIII restriction enzymes, followed by transferring the plasmid into *E. coli* JM109 competent cells using electroporation method. The positive pPRIMex30-ace2 clones of *E. coli* JM109 cells observed on the LB medium were again subjected to double digestion using *Xba*I and *Hin*dIII restriction enzymes. The results showed that the plasmid was cut into two major fragments (a) 1.4 kb of *ace*2 gene along with its terminator region (b) pPRIMex30 linearized fragments (Fig. 3a). The pPRIMex30-ace2 plasmids obtained were used for the construction of ace2-hph-PRIM plasmid using the linearized sequence Ppgk1-hph-Tcbh2 obtained from pTWO plasmid. The PCR amplification products of plasmid pTWO were retrieved and sequenced from 1% agarose gel, which showed a 2347 bp of Ppgk1-hph-Tcbh2 fragment. The Ppgk1-hph-Tcbh2 fragment was further inserted into linearized pPRIMex30-ace2 carrier using *Hin*dIII restriction enzyme. The circularized plasmid pPRIMex30-ace2 with Ppgk1-hph-Tcbh2 fragment were transferred into *E. coli* JM109 competent cells using electroporation method. The successful plasmids were further transformed into *T. reesei* QM9414 strain and cultured on growth medium supplemented with hygromycin. The *T. reesei* QM9414 observed on the culture plates was due to successful transformation and expression of hygromycin B phosphotransferase (*hph*) expression cassette, these positive strains were selected to validate the expression of constructed ace2-hph-PRIM plasmids. The double digestion of the plasmids with *Xba*I and *Hin*dIII restriction site has resulted in two fragments (a) 2.3 kb of hph expression cassette fragment (b) 4.8 kb of linearized pPRIMex30-ace2 carrier fragments on 1% agarose gel. These results have proved the successful construction of expression pace2-hph-PRIM vector (Fig. 3b). The *T. reesei* recombinant strains (positive ace2-hph-PRIM transformants) were also tested using colony PCR reaction to confirm the successful transformation insert gene and its size. We have isolated total of 7 recombinant strains out of which five recombinant strains *T*/Ace2-2, *T*/Ace2-5, *T*/Ace2-8, *T*/Ace5-4 and *T*/Ace10-1 were found to be stable and consistent. The genomic DNA of the above mentioned transformants was isolated and subjected to colony PCR reaction using *pki*-P1 and *ace2*-P6 primers (see Table 3). At about 100 bp from the transcriptional start site of the *ace2* upstream region in pace2-hph-PRIM recombinant plasmid and *pki*1 promoter upstream sequences and ace2 gene downstream region were used for designing the primers *pki*1 and *ace2*-P6. The PCR amplified fragments were further analyzed on 1% agarose gel electrophoresis. The results from electrophoresis shown in Fig. 4 confirms the successful transformation of ace2 recombinant plasmids. The successful transformants with ace2-hph-PRIM plasmids when amplified using the *pki*-P1 and *ace2*-P6 primers has resulted in 1.4 kb band for all the five positive transformants *T*/Ace2-2, *T*/Ace2-5, *T*/Ace2-8, *T*/Ace5-4 and *T*/Ace10-1 (Fig. 4: lane 2–6). Along with positive transformants, ace2-hph-PRIM plasmid was amplified using *pki*-P1 and *ace2*-P6 primers, which has also resulted in 1.4 kb band on the gel (Fig. 4). Along with the positive transformants and ace2-hph-PRIM plasmid DNA we have also used the *T. reesei* QM9414 genomic DNA as template for the PCR amplification reaction with the same primers (pki1-P1 and Ace2-P6) to confirm the specificity of the designed primers (*pki*-P1 and *ace2*-P6) towards the ace2-hph-PRIM plasmid. As expected *pki*-1 and *ace2*-P6 primers has failed to amplify the genomic DNA of *T. reesei* QM9414 (Fig. 4: lane 9).

**Fig. 5:**
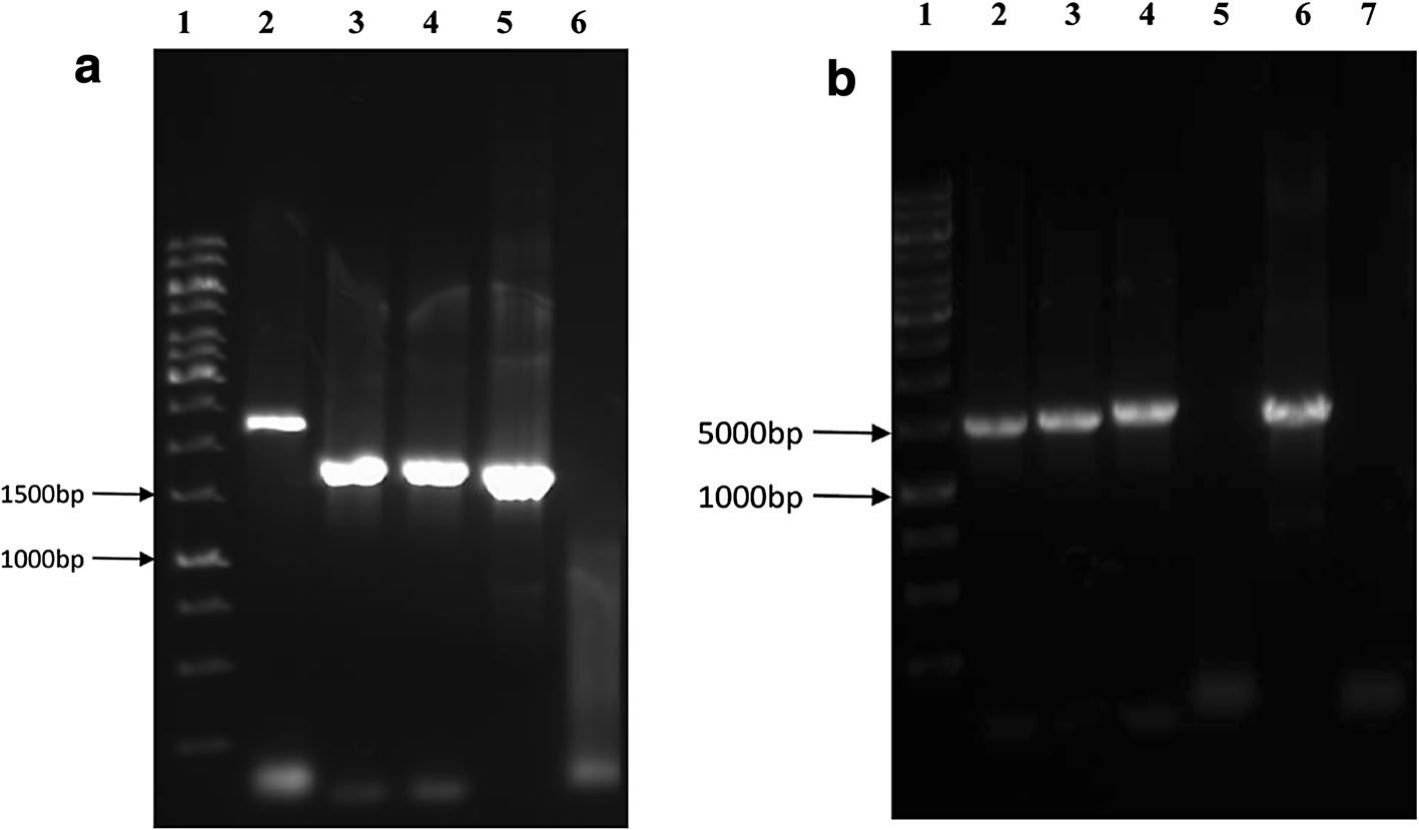
Shows the electrophoresis image of PCR amplified products of *ace2* gene from the genomic DNA of recombinant strains: **a** *Lane 1* 1 kb DNA marker; *Lane 2* recombinant strain *T/ace2*-*1*; *Lane 3* recombinant strain *T/ace5*-*4*; *Lane 4* recombinant strain *T/ace10*-*1*; *Lane 5* PCR amplified products of pace2-hph-PRIM plasmid (positive control); *Lane 6* PCR amplified products of *T. reesei* QM9414 genomic DNA (negative control). **b** *Lane 1* 1 kb DNA marker; *Lane 2* recombinant strain *T/ace2*-*2*; *Lane 3* recombinant strain *T/ace2*-*5*; *Lane 4* recombinant strain *T/ace2*-*8*; *Lane 5* recombinant strain *T/ace1*-*10*; *Lane 6* PCR amplified products of pace2-hph-PRIM plasmid (positive control); *Lane 7* PCR amplified products of *T. reesei* QM9414 genomic DNA (negative control)

Total of seven positive transformants were isolated after the gene transformation experiments, out of which five mitotic stable transformants were obtained and where further selected for the single spore isolation for obtaining the pure cultures. The five positive transformants were cultured for more than six generations, further the genomic DNA isolated from these positive transformants were further used as template for the PCR amplification using the pki1-P1 and Ace2-P6 primers (Table 3). The PCR amplified products were analyzed using 1% agarose gel electrophoresis, these results have shown a consistent band’s for all the positive transformants except for the ace2-1 and ace1-10 strains, where the PCR amplification resulted in no bands. The five stable positive transformants have showed consistent bands (Fig. 5a: lane 3–5, Fig. 5b: lane 2–4) similar as the positive control. The PCR amplification reaction with the recombinant strains ace2-1 and ace1-10 has failed to result in recombinant gene band.

### Cellulase and xylanase enzyme activities

**Fig. 6:**
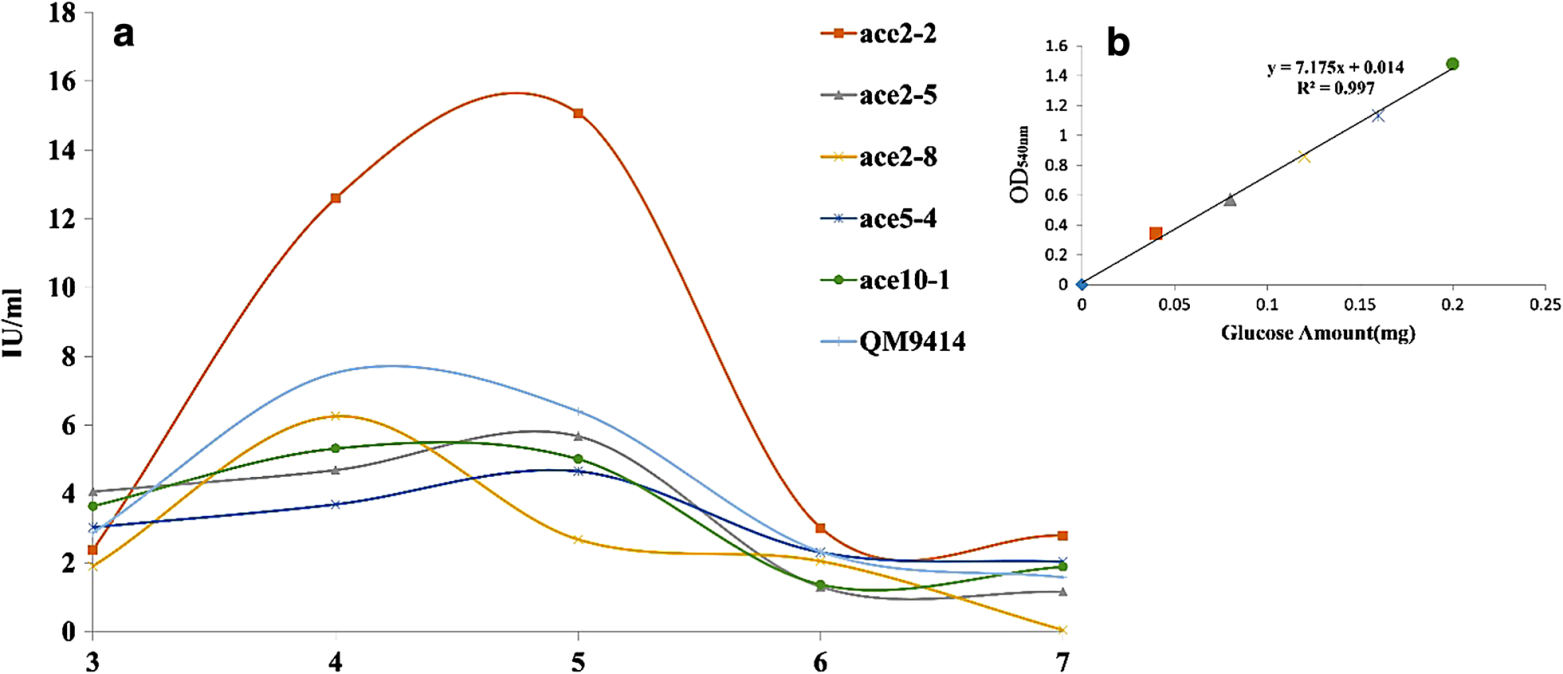
Compares the total cellulase activities (FPA) among recombinant strains *T*/Ace2-2, *T*/Ace2-5, *T*/Ace2-8, *T*/Ace5-4, *T*/Ace10-1 and the host strain *T. reesei QM9414*, **a** Total FPA activity, **b** Glucose standard curve

The five recombinant strains were further tested for their cellulase and xylanase enzyme activities. The recombinant strains were cultured on minimal salt medium supplemented with 2% crystalline cellulose cultivated for 168 h and the strains were sampled from 72 h (day 3). Filter paper assay was used for finding the total cellulase activities and MA-medium supplemented with 1% birch wood xylan was used for the total xylanase enzyme activities. The enzyme activities of the five recombinant strains (*T*/Ace 2-2, *T*/Ace 2-5, *T*/Ace 2-8, *T*/Ace 5-4, *T*/Ace 10-1) were compared with the parental strain (*T. reesei* QM9414). The enzyme activity of *T*/Ace 2-8, *T*/Ace 10-1 and *T. reesei* QM9414 show a rising trend between 72 and 96 h respectively (as shown in Fig. 6) and maximum FPA activity was found to be on 4th day with 6.27, 5.33 and 7.53 FPU/ml respectively. Similarly, FPA activity of *T*/Ace 2-2 and *T*/Ace 2-5 were found to be maximum at 120 h, with 5.69 and 4.67 FPU/ml respectively. Compared to other strains *T*/Ace 2-2 has showed a maximum total cellulase activity at 120 h (15.07 FPU/ml), with twofolds more than the maximum enzyme activity of QM9414 parent strain (Fig. 6). Based on the results obtained, declined FPA activity of recombinant strains after 120 h might be due to the altered growth conditions such as change in pH, autolysis and cell disruption of *T. reesei* strains.

**Fig. 7:**
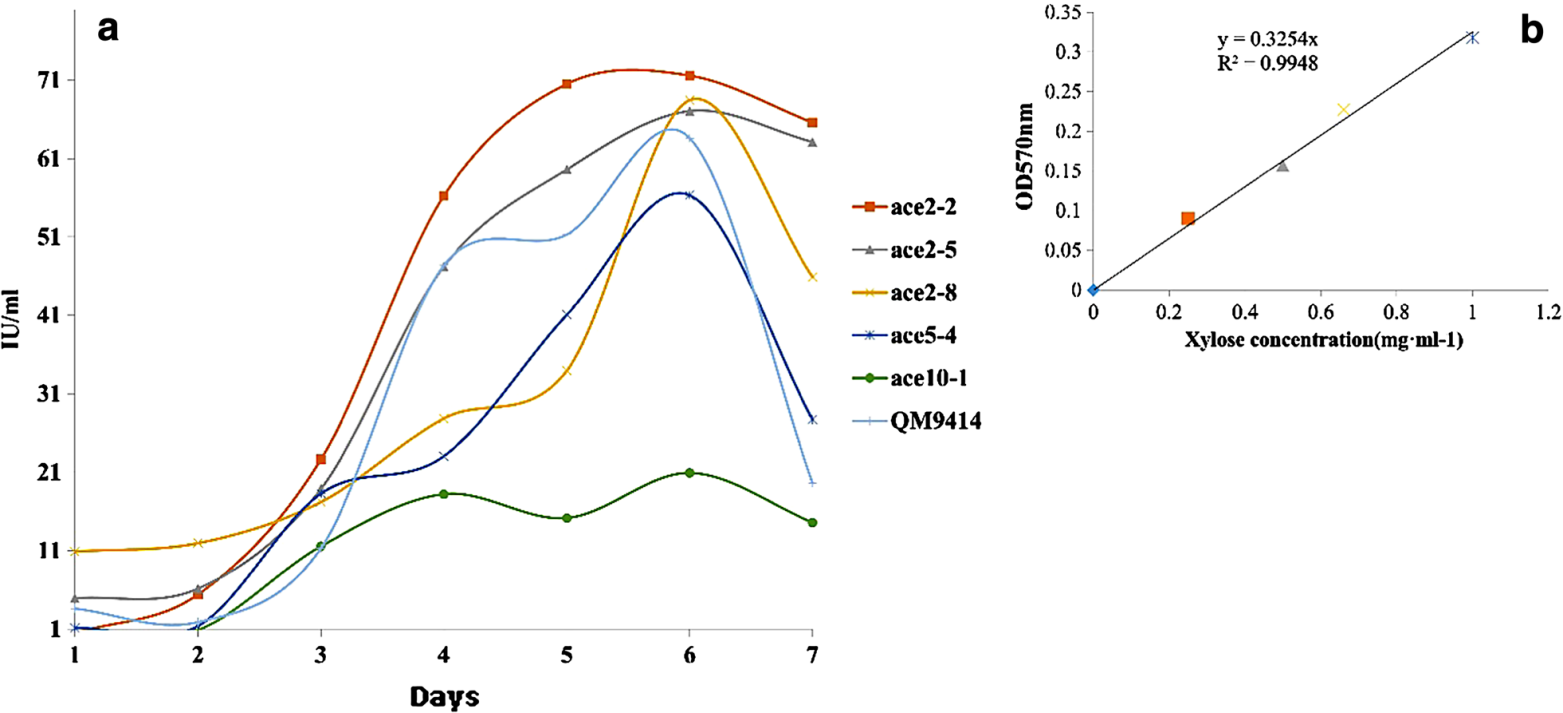
Compares the total xylanase activities among recombinant strains *T*/Ace2-2, *T*/Ace2-5, *T*/Ace2-8, *T*/Ace5-4, *T*/Ace10-1 and the host strain *T. reesei QM9414*, **a** Xylanase enzyme activity, **b** Xylose standard curve

The five recombinant strains were primarily cultured in MA medium containing 2% glycerol for 7 days at 30 °C and 180 rpm, 500 μl of culture samples were further used for the xylanase enzyme activities. Enzyme activity of the five recombinant strains were compared to the parent strain. The xylanase activity of the parent and recombinant strains increased from day 1 to day 6 in a sigmoidal pattern (Fig. 7). The maximum xylanase activity was exhibited by *T*/Ace2-2 from day 1 to day 6, by producing 71.56 IU/ml of xylanase by 6th day, the xylanase activity of *T*/Ace2-2 was found to be twofold high compared to parent strain. Followed by recombinant strains *T*/Ace2-5, *T*/Ace2-8 and *T*/Ace5-4 with 67.05, 68.44, 56.31 IU/ml respectively. Based on the results obtained, *T*/Ace2-2 strain exhibited overall two-fold higher cellulase and xylanase activities compared to *T. reesei* QM9414 strain. And *T*/Ace10-1 strain has shown low cellulase and xylanase activities compared to other strains (Tables 5, 6).

**Table 5:** Total cellulase activity of the recombinant strains and *T. reesei* QM9414 strain obtained from filter paper assay (FPA)

Cellulase-FPU/ml	Ace2-2	Ace2-5	Ace2-8	Ace5-4	Ace10-1	QM9414
Time (days)						
3rd	2.37	4.07	1.91	3.04	3.65	2.88
4th	12.60	4.70	6.27	3.70	5.33	7.53
5th	15.07	5.69	2.68	4.67	5.02	6.41
6th	3.02	1.31	2.05	2.31	1.37	2.33
7th	2.79	1.16	0.05	2.04	1.89	1.58

**Table 6:** Total xylanase activity of the recombinant strains and *T. reesei* QM9414 strain obtained from DNS assay

Xylanase (IU/ml)	Ace2-2	Ace2-5	Ace2-8	Ace5-4	Ace10-1	QM9414
Times (days)						
1st	0.50	4.92	10.90	1.15	0	3.61
2nd	5.41	6.15	11.97	1.39	0.9	1.89
3rd	22.62	18.85	17.21	18.27	11.56	11.31
4th	56.23	47.21	27.87	23.03	18.2	47.38
5th	70.49	59.59	33.94	41.06	15.16	51.31
6th	71.56	67.05	68.44	56.31	20.9	63.61
7th	65.58	63.12	45.9	27.71	14.59	19.59

### Estimation of xylitol produced from bark

**Fig. 8:**
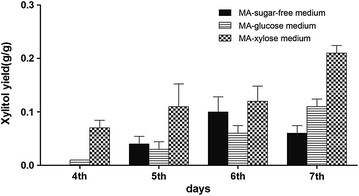
Shows the xylitol production yield from bark samples by *T*/Ace2 recombinant strain, cultured in MA growth medium supplemented with simple sugars (glucose and xylose) and MA-sugar free growth medium for 7 days

The efficiency of the *ace2* recombinant strains for the production of the xylitol is estimated by culturing the strains in MA-medium supplemented with 2% bark samples. Based on the results obtained from the cellulase and xylanase enzyme activities we have selected *T/*Ace2-2 for testing its xylitol production efficiency. The xylitol production yield by the *T/*Ace2-2 strain was estimated using High-Performance Anion Exchange Chromatography with Pulsed Amperometric Detection (HPAEC-PED). The growth medium used for the estimation of xylitol production can be classified into MA-sugar (d-glucose, d-xylose) medium and MA-sugar free medium, to see the effect of simple sugars in xylitol production. Results obtained from HPAEC show that under MA-sugar free conditions, xylitol production reached the maximum by *T*/Ace2 strain with 4.77 g/l on 6th day, with a conversion rate of 0.1 g/g. The xylitol production yield by *T*/Ace2 strain slightly increased to 5.34 g/l on 6th day when cultured in MA-d-glucose growth medium. A significantly high xylitol production yield was observed when *T*/Ace2 strain was cultured on MA-medium supplemented with d-xylose with 10.52 g/l on 7th day of the cultures with a conversion rate of 0.21 g/g (Fig. 8). Whereas xylitol yield was found to be decreased in MA-sugar free and MA-d-glucose growth mediums with 3.18 and 3.21 g/l respectively (Table 7).

**Table 7:** Shows the total xylitol produced through degradation of bark by recombinant strain T/Ace2-2 using HPAEC

$\frac{{C xylitol\,({\text{g/l}})}}{{Y xylitol\,({\text{g/g}})}}$	MA-sugar-free medium	MA-glucose medium	MA-xylose medium
Time (days)			
4th	0.19 ± 0/0.00	0.61 ± 1.18/0.01	3.60 ± 1.27/0.07
5th	1.84 ± 1.29/0.04	1.75 ± 2.11/0.03	5.25 ± 3.52/0.11
6th	4.77 ± 2.63/0.10	5.34 ± 2.17/0.06	5.89 ± 2.62/0.12
7th	3.18 ± 2.17/0.06	3.21 ± 1.97/0.11	10.52 ± 1.82/0.21

### SEM analysis of degrading bark T/Ace2-2 strain

**Fig. 9:**
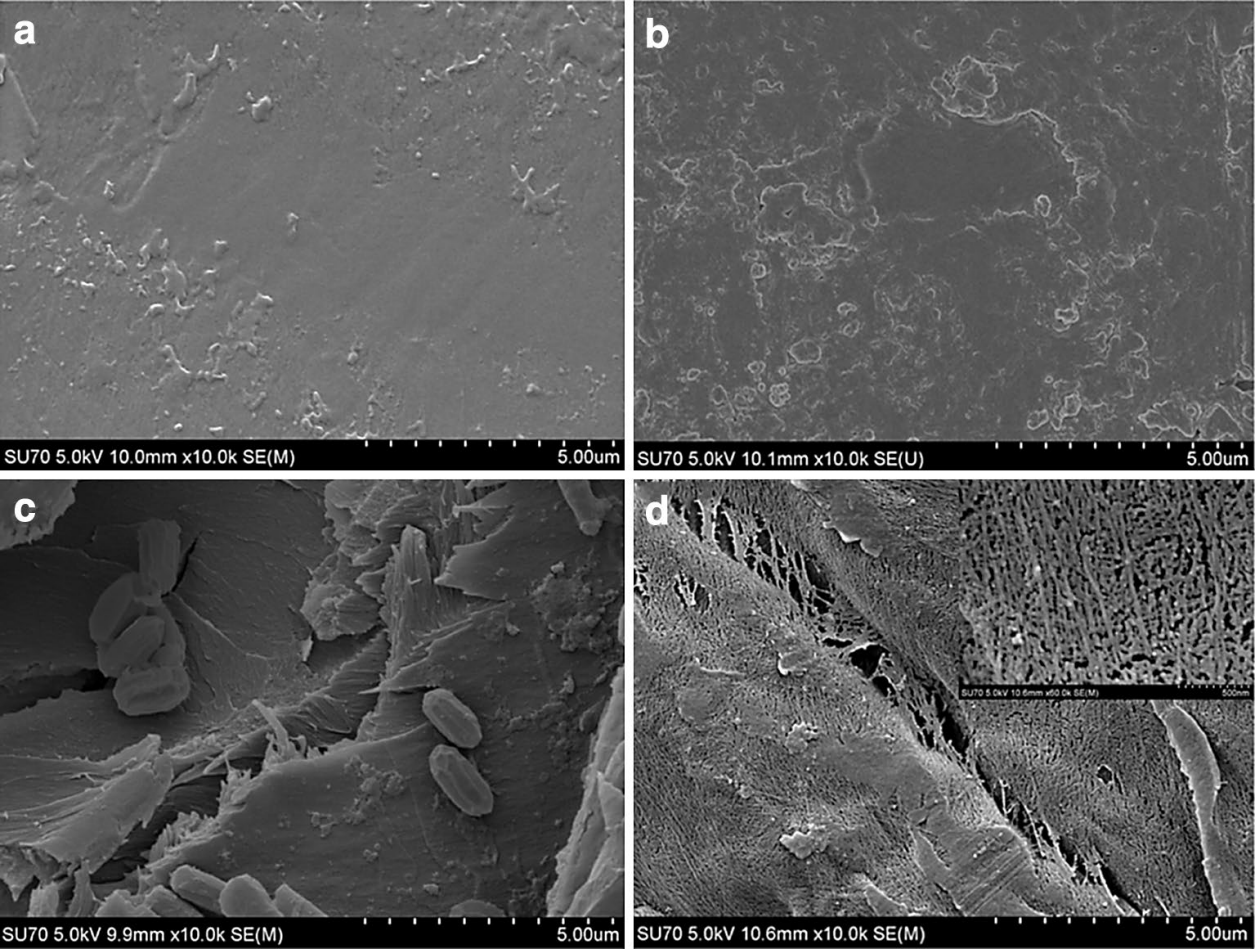
Scanning electron microscopy (SEM) images of the tree barks. The untreated bark samples (control) within an incubation period of **a** 14 days and **b** 28 days. **c** The bark samples treated with T/Ace2-2 recombinant strains, within an incubation period of **c** 14 days and **d** 28 days

In order to understand the molecular level changes occurred in the bark samples we have performed a scanning electron microscopy analysis of *T/*Ace2-2 inoculated bark samples. The bark samples were cut into small pieces; few pieces were dropped into *T/*Ace2-2 strain free 50 ml MA medium and incubated at 30 °C and 200 rpm for 28 days. Similarly, few bark pieces were dropped into flasks containing *T/*Ace2-2 inoculated 50 ml MA medium and incubated at same conditions used for the control. The SEM images were taken for both the control (*T/*Ace2-2 free) samples and test samples on 14 and 28 days respectively (Fig. 9). As expected the bark samples incubated in *T/*Ace2-2 containing MA medium has showed progressive breakdown of the bark cells. The SEM images of 14-day bark samples (Fig. 9c) shows large number of fungal cells growing inside the bark layers including the epidermis with vascular bundle sheath and soft tissue cells found to be degraded. We have also observed that 14-day bark cultures exhibited a loosened surfaces and visible lacuna. From the SEM images of 28-days bark cultures, we can observe that large number of cells were found to be degraded with visible fractures and cavities in the bark. These images also show degraded bark samples with exposed microfibers and complex interwoven net structures (Fig. 9d). The 14-day and 28-day *T/*Ace2-2 free bark cultures were found to occur in a smooth sheath without any visible cavities or fractures (Fig. 9a, b). The SEM images of 14-day and 28-day bark samples reveal that the recombinant strain *T/*Ace2-2 was able to depolymerize the bark cellular complex by colonizing the inner cell walls such as epidermis.

## Discussion

Research studies conducted in the past on microbial cellulose degradation have revealed that five transcription factors such as XYR1, ACE2, HAP2/3/5, ACE1 and CRE1 were involved in expression of cellulase encoding genes [49]. All these transcription factors work in a consistently with a specific fine-tuned mechanism. Aro et al. [24] have reported that neither ACE2 nor ACE1 transcription factors are responsible for the expression of cellulase and hemicellulases encoding genes in *T. reesei* [24]. Among the above mentioned transcription factors XYR1 (xylanase regulator 1) is considered as the important activator of cellulase and hemicellulase encoding genes [49]. ACE2 is the main coactivator, and its influence is specific for cellulose based growth of *T. reesei* and other cellulolytic fungi [24]. Several research studies were conducted in the past to increase the output of a target protein by increasing the number of gene copies, which was fund to be successful in *Aspergillus* spp. and *Trichoderma* spp. While many experiments have proved that when the copy number reached a certain value, the target protein does not increase with an increase in copy number. The lack of certain regulatory proteins or some key transcription regulator factors causes this difference in target protein expression [51, 52]. Gene expression and regulation studies conducted on filamentous fungi have suggested that, most of the genes encoding for the cellulose and hemicellulose degrading enzymes were majorly regulated during transcriptional stages [53].

Our present study was focused on increasing the expression of lignocellulose degrading enzymes in *T. reesei* QM9414 strain. Gene sequence coding for the transcription regulatory factor *ace2* was retrieved and was cloned from *T. reesei* QM9414 and later successfully expressed using an expression vector pace2-hph-PRIM. Kurzatkowski et al. [54] has successfully constructed a xylanase recombinant strains carrying xyn1 and xyn2 structural genes with *pki1* (pyruvate kinase encoding) promoter to produce xylanases with *T. reesei* cultured on glucose. The successful transformants exhibited xylanase activities of 76 (XYN I) and 145 U/mg (XYN II) when grown on glucose respectively [54]. The expression vector reported in our present study (pace2-hph-PRIM) carries *pki1* promoter for regulating the expression of target genes. We have found 7 positive transformants from our transformation experiments, these recombinant strains were further isolated using single spore and were further subcultured to attain stable strains. Finally, we have retrieved five stable recombinant strains *T*/Ace2-2, *T*/Ace2-5, *T*/Ace2-8, *T*/Ace5-4 and *T*/Ace10-1. The filter paper assay and xylanase enzyme activities of these five recombinant strains have been determined. From the results obtained we have found that the total cellulase and xylanase activities of *T*/Ace2-2 and *T*/Ace2-5 recombinant strains are significantly higher than the maximum enzyme activity of parent strain *T. reesei* QM9414 strain. By sixth day, the xylanase activity of *T*/Ace2-2 is 1.12 times more than QM9414 strain, similarly total cellulase activity of *T*/Ace2-2 is three times more than QM9414 strain. The increased expression of the cellulase and xylanase in the *T*/Ace2-2 recombinant strain can be attributed to the expression of transcription regulatory factor *ace2,* as it is involved in the regulation of cellulase and hemicellulase expression [55]. According to Aro et al. [24], the deletion of *ace2* transcription factor has resulted in lowered transcription levels of cellulases and reduced the overall cellulase activity by 30–70% [24].

The recombinant strain *T*/Ace2-2 was further used for the degradation of bark to produce xylitol. Valentín et al. [56] have reported the bark composition of *Pinus sylvestris* and used pine bark for the bioremediation purposes using *Phanerochaete velutina* and *Stropharia rugosoannulata* cultures. This study has reported that original pine bark contained 45% lignin, 25% cellulose and 15% hemicellulose, both the fungal strains *P. velutina* and *S. rugosoannulata* were able to degrade all the components of the bark [56]. The mycelium of *T*/Ace2-2 recombinant strain was inoculated in 2% bark-sugar-free-MA medium, 2% bark-MA-glucose medium and 2% bark-MA-xylose medium respectively. The xylitol production yields of 2% bark-sugar-free-MA medium and 2% bark-MA-glucose medium didn’t increase obviously. However, after adding d-xylose into MA medium, conversion rate of xylitol from degraded bark increased significantly. By 7th day, we have found that xylitol conversion rate of 2% bark-MA-xylose medium was 10.52 g/l with a productive rate of 0.21 g/g. Prathumpai et al. [57] reported that xylitol production yields of *Aspergillus nidulans* remarkably raised when cultured in fermentation medium supplemented with d-xylose and when d-glucose was supplemented to the fermentation medium xylitol yield does not increase significantly [57]. Finally, the results obtained from the SEM analysis have confirmed the degradation of bark by *T/*Ace2-2 recombinant strain, by exposing the inner microstructure of the degraded bark, whereas the bark samples without *T/*Ace2-2 inoculation was found to exhibit a closed and intact surface morphology. Lourenço et al. [58], have performed the pyrolysis gas chromatography for analyzing the sapwood and heartwood barks of 70 year old teak trees [58]. This study has revealed that high lignin content of 35.4 and 37.3% was observed for sap and heart wood teak, teak wood categorized as GS type of lignin containing G (56.0%), S (42.2%) and H (1.8%) residues with a G/S ratio of 0.8 [58]. From the SEM results it can be inferred that recombinant *T/*Ace2-2 strain could successfully disrupt the intricate lignocellulosic networks and utilize hemicellulose and cellulosic units for its growth and metabolism and the xylitol production yields also confirm this fact. Dashtban et al. [59] have conducted double gene deletion experiments with xylitol dehydrogenase and l-arabinitol-4-dehydrogenase to breakdown the preprocessed barley straw for the production of xylitol [59]. The maximum xylitol production yield reported by Dashtban et al. was 13.2 g/l, which was higher than the xylitol conversion efficiency of our study. The major reason behind the lower conversion efficiency might be due to the use of direct bark samples without any pretreatment for the microbial degradation performed in our current study. While the maximum xylitol production yield reported by Hong et al. [60] was 3.7 g/l was found to be lesser than the production yield reported in our current study. Hong et al. [60] have selected mixed carbon source containing xylose and glucose for the production of xylitol [60]. In our present work, we have used a novel, cost effective and eco-friendly bio-transformation method for the production of xylitol using bark as a feedstock, to improve the comprehensive utilization of resources and development of circular economy. The efficient total cellulase and xylanase activities of the recombinant strains can be applied in the field of bio refinery for the efficient conversion of cellulose and hemicellulose containing plant biomass for the production of biofuels and other commercially valuable products.
